# Incidence and Pattern of Dry Eye after Cataract Surgery

**DOI:** 10.1371/journal.pone.0078657

**Published:** 2013-11-12

**Authors:** Ngamjit Kasetsuwan, Vannarut Satitpitakul, Theerapa Changul, Supharat Jariyakosol

**Affiliations:** 1 Department of Ophthalmology, Chulalongkorn University, Bangkok, Thailand; 2 Department of Ophthalmology, Chainat Hospital, Chainat, Thailand; Medical University Graz, Austria

## Abstract

**Purpose:**

To evaluate the incidence and severity pattern of dry eye after phacoemulsification.

**Setting:**

King Chulalongkorn Memorial Hospital, Bangkok, Thailand.

**Design:**

Prospective descriptive study.

**Methods:**

Samples were collected from ninety-two uncomplicated cataract patients who were 18 years old or older. Dry eye incidence and pattern were analyzed at days 0, 7, 30 and 90 after phacoemulsification using (1) Ocular Surface Disease Index (OSDI) questionnaire, (2) tear break up time (TBUT), (3) Oxford ocular surface staining system, and (4) Schirmer I test without anesthesia.

**Results:**

Seven days after phacoemulsification, the incidence of dry eye was 9.8% (95% confidence interval; 3.6–16.0%). The severity of dry eye peaked seven days post-phacoemulsification and was measured by OSDI questionnaire and all three clinical tests. Within thirty days and 3 months post-surgery, both the symptoms and signs showed rapid and gradual improvements, respectively. However, dry eye post-phacoemulsification was not significantly associated with sex and systemic hypertension (P = 0.26, 0.17 and 0.73, respectively).

**Conclusions:**

The incidence of dry eye after phacoemulsification was 9.8%. Symptoms and signs of dry eye occurred as early as seven days post-phacoemulsification and the severity pattern improved over time. We recommend that ophthalmologists should evaluate patients both before and after phacoemulsification to prevent further damage to the ocular surface and able to manage the patient promptly and effectively so the patient will not have a poor quality of life and vision due to dry eye syndrome.

## Introduction

Dry eye is a multifactorial disease of the tears and ocular surface. [1] Ocular symptoms such as pain, irritation, and poor vision can result from dry eye. Severe dry eye affects the patient’s ocular and general health, well-being, and quality of life, [1]–[3] Since the dry eye syndrome is common worldwide, it can be caused by many things. Numerous epidemiologic studies [4]–[8] have reported that aging, connective tissue disease, history of allergy or diabetes, and use of antihistamines and refractive surgery are risk factors for the development of dry eye syndrome.

Many patients who have undergone cataract surgery, the most common procedure performed in ophthalmic units, have complained of dry eye and symptoms of irritation postoperatively. Complications such as dry eye syndrome can occur after an extracapsular cataract extraction because a large incision is created in the eye during the procedure that sometimes damages the cornea. [9] Phacoemulsification is also commonly performed worldwide; a smaller incision is created and ultrasonic-driven oscillating tips are used to emulsify or fragment the crystalline lens. Few reports of dry eye syndrome have focused on patients who had undergone phacoemulsification and subsequently developed dry eye.

In the current study, we used various measurements to assess the incidence and severity pattern of dry eye syndrome among patients who have undergone phacoemulsification.

## Materials and Methods

This prospective, descriptive study was conducted at the outpatient and surgical unit of Department of Ophthalmology, King Chulalongkorn Memorial Hospital, Bangkok, Thailand, from 2010 to 2011. Ninety-two patients 18 years or older with a cataract and no dry eye, as assessed by the Ocular Surface Disease Index (OSDI) questionnaire (OSDI scores of 25 or less), were recruited. Patients who received concomitant medications that could cause dry eye such as antihistamines, antidepressants, birth control pills, decongestants, medications in the accutane, gabapentin, sildenafil citrate, anticholinergic drugs and who had autoimmune diseases were excluded. Patients were also excluded if they developed complications during phacoemulsification.

### Ethics Statement

The Institutional Review Board, Faculty of Medicine, Chulalongkorn University, approved and monitored this study. All patients provided written informed consent before starting the study.

The sample size in this study was calculated based on the study of Lekhanont et al. [10] which reported 34% prevalence of dry eye in 550 patients in Bangkok. The incidence of dry eye was assessed using the OSDI questionnaire. The severity pattern of dry eye after phacoemulsification was obtained from the average of the OSDI scores, tear break-up time (TBUT), the Schirmer I test without anesthesia and the Oxford Schema. The patients’ baseline and postoperative characteristics were recorded. The incidence of dry eye on day 7 after phacoemulsification was assessed using the OSDI questionnaire, [11] a 12-item questionnaire used worldwide to accurately assess symptoms of ocular irritation related to dry eye and vision. We modified the questionnaire by omitting items 4 and 5, which assess the presence of blurred and poor vision, because it is difficult to differentiate the change of these symptoms caused by cataract surgery alone or combined with visual symptoms due to cataract surgery induced dry eye conditions. The total OSDI score was calculated using the following formula:

OSDI score = (sum of all answered questions) x 100/total number of answered questions) x 4.

The OSDI scores range from 0 to 100. Scores from 0 to 25 are considered normal; scores exceeding 25 indicate the presence of dry eye symptoms.

After completing the questionnaire, the following tests were performed consecutively: TBUT, fluorescein staining with Oxford Schema, and Schirmer I test without anesthesia. The TBUT [1] measures the interval between the last complete blink and the first appearance of a dry spot or disruption of the tear film. Three TBUT scores were averaged to determine whether the patient had dry eye. An average score of 10 seconds or more was classified as normal; a TBUT shorter than 10 seconds indicated the presence of dry eye. Conjunctival and corneal fluorescein staining were graded using the Oxford Schema, [1] with 0 to I indicating normal and II to V indicating dry eye. For the Schirmer I test [1] without anesthesia, Schirmer paper strips were inserted over the lower lid margin, midway between the middle and outer third of the lid. The wetness on the strip was measured 5 minutes after application. A wet area that measured 10 mm or less was diagnosed as dry eye. The OSDI questionnaire and the three clinical tests were administered on day 0 (baseline), week 1, and months 1 and 3 after phacoemulsification.

One surgeon (N.K.) performed all cataract surgeries with the patients under topical anesthesia induced with 0.5% tetracaine hydrochloride. Before surgery, the eye was prepared and draped using sterile techniques. Phacoemulsification was performed with a 2.75-mm temporal clear corneal incision and a side port of about 1 mm 90-degree incision away from the main incision. The range of phacoemulsification time was 5 to 10 minutes and foldable intraocular lens were inserted thereafter. There was no intraoperative complication in all cases. After surgery, all patients instilled Tobradex® (tobramycin with dexamethasone ophthalmic, Alcon, Fort Worth, TX) eye drops four times daily for 1 month.

The dry eye pattern detected by the mean OSDI, TBUT, and Schirmer I test without anesthesia scores were analyzed as continuous data and by the Oxford ocular surface staining system as it was an ordinal data. Kappa analysis was used to assess the agreement among the four tests. The associations between age, sex, underlying disease, and dry eye postoperatively were analyzed by Fisher’s exact test. All statistical analyzes were performed using Stata software, version 11 (StataCorp. 2009. *Stata Statistical Software: Release 11*. College Station, TX: StataCorp LP.) An alpha value of 0.05 was considered significant.

## Results

Data from 92 eyes of 92 patients (one eye of each patients) were analyzed. The demographic patient data are shown in Table 1. Sixty-six percent of the patients were women (mean age, 67.22±8.26 years).

**Table 1:** Demographic data.

			Case (n = 92)
Sex, n (%)			
	Male		31 (33.70%)
	Female		61 (66.30%)
Age (years)			
	Mean ± SD (range)		67.22±8.26 (42–84)
Underlying disease, n (%)			
	Hypertension		48 (52.17%)
	Dyslipidemia		19 (20.65%)
	Diabetic Mellitus		18 (19.57%)
	Heart disease		9 (9.78%)
	Glaucoma		3 (3.26%)
	Benign prostatic hypertrophy		3 (3.26%)
	Other (Parkinson, HBV, asthma, hyperthyroid)		<3%

On days 7, 30, and 90, data from the OSDI questionnaire and three clinical tests were available from 92 (100%), 82 (89.1%) and 74 (80.4%) patients, respectively. On day 7, nine of 92 cases had OSDI scores exceeding 25. The incidence of dry eye was 9.8% (95% CI, 3.6%–16.0%). The changes in the other tests corresponded to the OSDI results (Table 2).

**Table 2:** Incidence of dry eye at day 7 after phacoemulsification.

Test	Incidence (95% CI)
OSDI	9.8% (3.6–16.0)
TBUT	68.4% (52.9–83.9)
Oxford Schema	58.7% (47.2–70.1)
Schirmer I without anesthesia	11.9% (3.4–20.4)

On day 7 postoperatively, the mean scores of OSDI questionnaire (preoperatively vs. postoperatively, 12.57 vs. 33.87, respectively), TBUT (preoperatively vs. postoperatively, 12.15 vs. 4.59 seconds), Oxford Schema (preoperatively vs. postoperatively, grade 1 vs. 2), and Schirmer I without anesthesia (preoperatively vs. postoperatively, 14.14 vs. 7.57) showed a trend toward dry eye syndrome. The agreement between the three clinical tests and the OSDI questionnaire in various combinations was analyzed by Kappa analysis and showed no agreement with each other except for the OSDI questionnaire with the Oxford Schema and the TBUT with the Oxford Schema. Poor agreement was detected between the OSDI questionnaire and the Oxford Schema (agreement 47.2%, kappa 9.8%, p = 0.03); the TBUT and the Oxford Schema (agreement 73.0%, kappa 39.9%, p<0.001) showed fair agreement.

**Figure 1:**
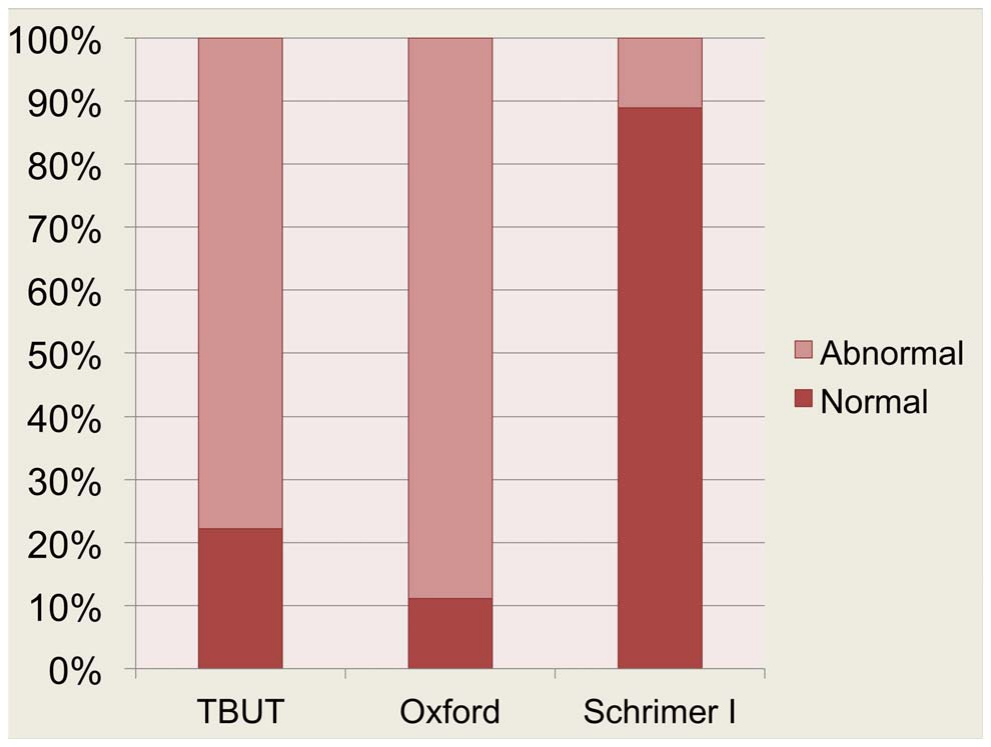
Percentage of patients with/without signs who reported OSDI symptoms. The results between the presences of symptoms assessed by the OSDI questionnaire in combination with one of the clinical tests. Patients with OSDI symptoms often had decreased TBUT (77.78%) and abnormal Oxford Schema (88.89%) scores in contrast to Schirmer I test without anesthesia. According to the Schirmer I test without anesthesia, 88.89% had a normal test.

Figure 1 shows the results between the presences of symptoms assessed by the OSDI questionnaire in combination with one of the three clinical tests on day 7. Patients with OSDI symptoms often had a decreased TBUT (77.78%) and an abnormal Oxford Schema (88.89%) scores in contrast to the Schirmer I test without anesthesia. According to the Schirmer I test without anesthesia, 88.89% had a normal test.

**Figure 2:**
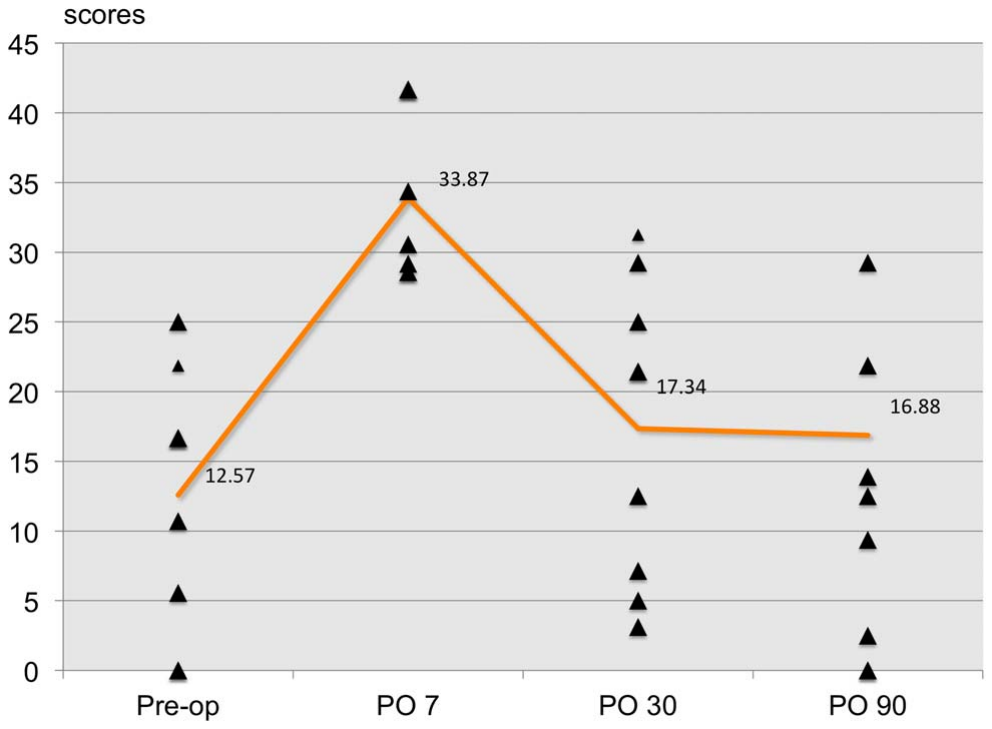
Pattern of dry eye after post-operation according to the OSDI scores. The mean OSDI scores at day 7, months 1 and 3 after surgery were 33.87, 17.34 and 16.88 scores respectively.

**Figure 3:**
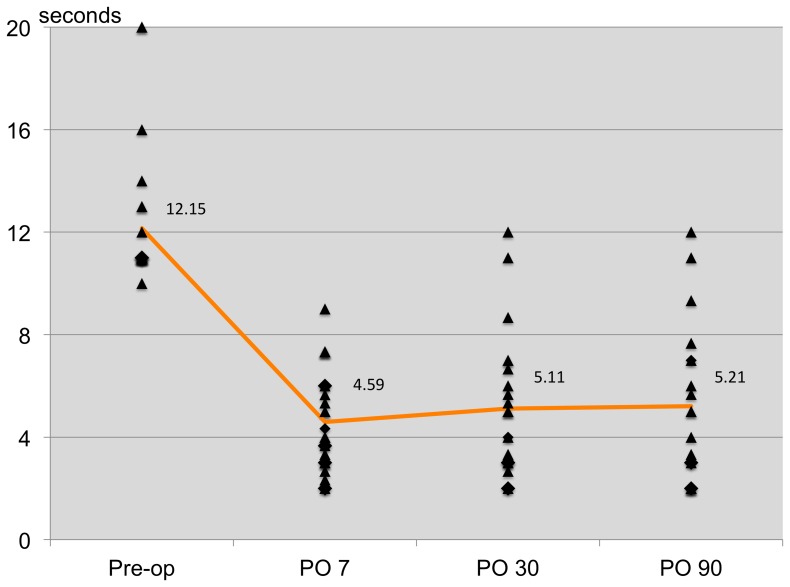
Pattern of post-operative dry eye patients detected by TBUT.

**Figure 4:**
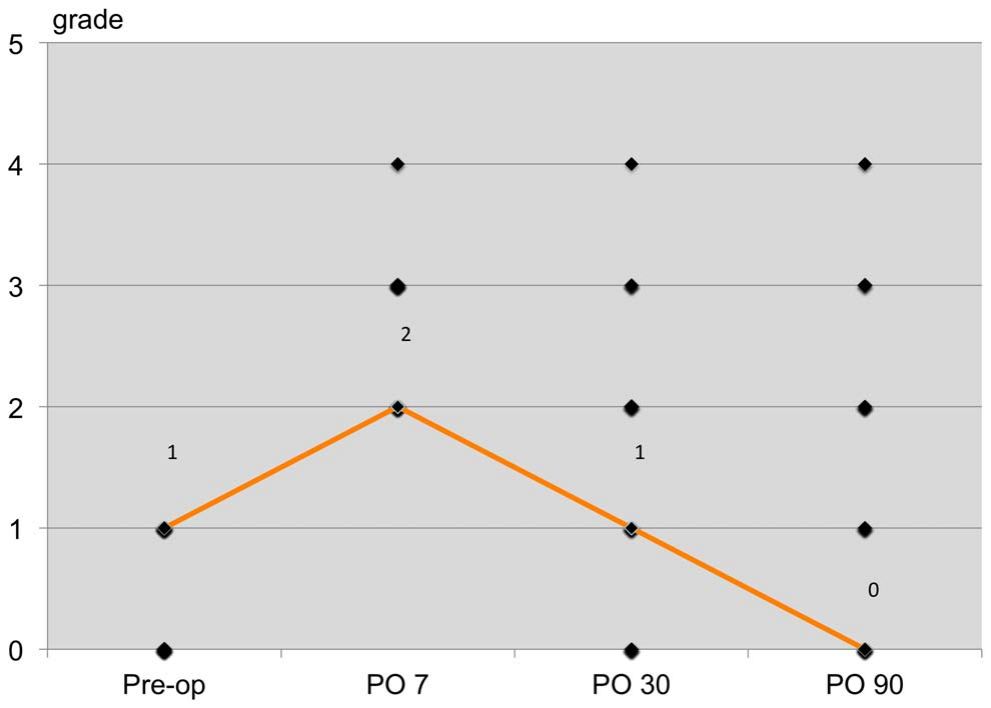
Pattern of post-operative dry eye patients detected by Oxford ocular surface staining system.

**Figure 5:**
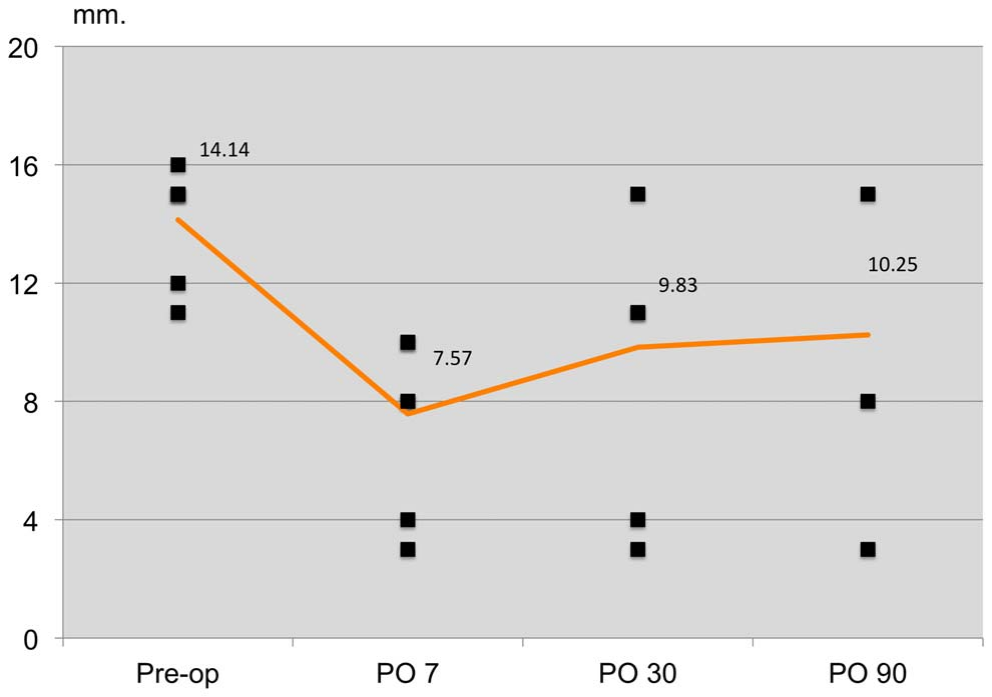
Pattern of post-operative dry eye patients detected by Schirmer I without anesthesia. The patterns of dry eye were similar between 3 clinical tests with OSDI questionnaire.

The patterns of dry eye postoperatively based on the OSDI scores are shown in Figure 2. The mean OSDI scores on day 7 and months 1 and 3 postoperatively were 33.87, 17.34, and 16.88, respectively. The dry eye patterns were similar between all three clinical tests and the OSDI questionnaire (Figs 3–5).

Only four of nine patients with dry eye postoperatively had systemic hypertension. No patient had other underlying diseases. No correlations were found between postoperative dry eye and sex (p = 0.26), age (p = 0.17), or systemic hypertension (p = 0.73).

## Discussion

Dry eye is a multifactorial disease of tears and ocular surface that can be result from aqueous deficiency or be evaporative in nature. This syndrome affects individuals worldwide. Long-term population-based studies have shown the incidence rates of dry eye among the population between ages 43 and 86 years at 5 and 10 years of follow-up to be 13.3% and 21.6% respectively. [4], [5] In Thailand, the incidence of dry eye in a hospital-based population was 34%. [10].

Dry eye can develop often after various types of ophthalmic surgeries such as photorefractive keratectomy and laser-assisted in situ keratomileusis (LASIK). The incidence rates of dry eye, assessed by corneal fluorescein staining 1 week postoperatively for either nasal- or superior-hinge LASIK, were 47.06% and 52.94%, respectively. [6] In addition, after LASIK, dry eye can persist for up to 6 months or more with an incidence of 20%, [7] whereas in patients who have undergone blepharoplasty, dry eye can last up to 2 weeks or more with an incidence of 10.9%. [12] Even though many previous studies have compared the preoperative and postoperative changes in dry eye symptoms and/or the dry eye test values that worsened significantly after cataract surgery, [9], [11], [13] the current study was the first to report the incidence and pattern of dry eye after phacoemulsification using various combinations of tests over a 90-day period.

A difficulty in assessing dry eye is that there is no gold standard test. [1] As a result, various diagnostic tools with different sensitivities and specificities are used to diagnose dry eye. Questionnaire is often used in epidemiologic research studies to assess dry eye. [1] In the current study, we used the OSDI questionnaire because it reliably assesses the severity, natural history, and effects of dry eye. Compared to the Short Form 12-Health Survey, the National Eye Institute Visual Functioning Questionnaire, and the McMonnies Dry Eye Questionnaire, the OSDI questionnaire has a sensitivity of 60% and specificity of 79%. [12] We modified the OSDI by omitting two of the five ocular symptom-evaluation questions pertaining to blur and poor vision so we believe that we can avoid bias due to better visual acuity postoperatively.

Despite of Rose Bengal is more sensitive to evaluate the ocular surface in dry eye patients, we only reported the result of corneal and conjunctival fluorescein staining since most patients denied using Rose Bengal staining postoperatively because of discomfort caused by the stain.

The reason why the TBUT and Oxford Schema indicated more cases postoperatively were compared to the Schirmer I test without anesthesia (Table 2) is because these tests can easily detect tear film instability and ocular surface inflammation respectively Abnormal TBUT and Oxford Schema may result from microscopic light exposure, toxic substances from inflammatory cytokines, medications, or preservatives. Despite of only mild injected bulbar conjunctiva and no significant anterior segment inflammation found postoperatively, high percentage of patients developed abnormal TBUT and Oxford Schema. Phacoemulsification can affect or interrupt the neurogenic response of the ocular surface and decrease tear secretion. However, the small number of cases with an abnormal Schirmer I test without anesthesia indicated that phacoemulsification affects the tear film stability and ocular surface inflammation more than tear secretion.

Like other studies, we also have reported that dry eye can develop after cataract surgery. [14]–[17] Li et al. [14] reported high percentages of patients who developed dry eye symptoms after phacoemulsification, lower tear meniscus height, decreased TBUT scores, decreased Schirmer I test scores, and serious squamous metaplasia detected by impression cytology. Liu et al. [15] also reported significant worsening of the tear film pattern, height of the tear meniscus, and scores detected by the TBUT, Schirmer I test, and corneal fluorescein staining after phacoemulsification. Barabino et al. reported that phacoemulsification induced dry eye-like symptoms and signs on days 1 and 7 (unpublished data presented at the 6^th^ International Conference on the Tear Film and Ocular Surface, Florence, Italy, September_2010). In one case series, 10 patients (12 eyes) presented with severe keratopathy including epithelial keratopathy, central epithelial ulcer, and central stromal ulcer after cataract surgery. [16] In contrast, Ram et al. [17] reported no differences in dry eye between before and after phacoemulsification in 23 patients when the TBUT and Schirmer I test with anesthesia were performed. The reason for the discrepancy may be due to its small sample size and retrospective study design.

Regarding the pattern of postoperative dry eye, our findings were consistent with the results of Barabino et al. (unpublished data) who detected dry eye on the seventh day after surgery and rapid improvement within 30 days postoperatively. We did not found any late reaction of dryness such as filamentary keratopathy, superior limbic keratoconjunctivitis or persistent epithelial defect. However, one study reported that dry eye symptoms and a lower tear meniscus developed at 1 month and continued for another 2 months. [14] A possible explanation for this difference may be due to the different topical ocular drops and duration of regimens used: Tarivid Ophthalmic Solution (ofloxacin, Daiichi Sankyo, Tokyo, Japan) 4 times daily for 2 weeks, Pred Forte (prednisolone acetate ophthalmic suspension, USP 1%, Allergan, Irvine, CA) 4 times daily for 1 week, and Pranopulin Eye Drops (Senju Pharmaceutical, Chuo-Ku, Osaka, Japan) 4 times daily for 1 month. The surgical techniques may have affected their results, in which neither the size nor location of the wound was reported.

Another explanation for the dry eye pattern observed in the current study was the recovery process of the corneal nerves. Since the cornea is one of the most highly innervated organs, with about 44 corneal nerve bundles entering the cornea around the limbus centripetally [18] and larger nerve fibers that run from the 9 o’clock to the 3 o’clock position and bifurcate to achieve a homogenous distribution over the entire cornea, [19] it is vulnerable to any damage within that region. Temporal corneal incisions created during phacoemulsification can reduce the corneal sensitivity in the surgical area and other areas far from the incision site. [20], [21] The damage to the corneal nerves may expand when longer phacoemulsification time is needed to break up a dense cataract. [21] Neurogenic inflammation also can develop after corneal incisions. Inflammatory mediators can change the action of the corneal nerves and reduce corneal sensitivity. [22] Disruption of the normal corneal innervation or lacrimal functional unit feedback can reduce the tear flow and blink rate and cause instability of the tear hyperosmolarity and tear film. [1] With corneal healing postoperatively, new neurite cells emerge and after 25 days, neural growth factor is released to regenerate the subepithelial corneal axon. [22] Thus, the recovery of the corneal nerves may explain why the dry eye was seen early after surgery and improved thereafter. Even though, in theory, neurogenic inflammation may effect by feedback loop to contralateral eye, we did check the other eye as in general as screening and did not find any significant dryness developed after surgery.

In addition to transection of the corneal nerves and damage to the corneal epithelial cells, exposure to microscopic light, vigorous intraoperative irrigation of the tear film, elevation of inflammatory factors in the tear film due to ocular surface irritation, use of topical anesthesia intraoperatively, and topical eye drops administered postoperatively and its preservatives can cause dry eye after phacoemulsification. [11], [14], [16], [23], [24] Vigorous irrigation of the tear film and manipulation of the ocular surface intraoperatively may reduce the goblet cell density and result in shortened TBUT postoperatively. [14] We believe that the use of light filters, decreased exposure time, appropriate irrigation, and gentle handling of the ocular surface tissue may decrease the postoperative complications.

Moreover, benzalkonium chloride, one of the most commonly used preservatives in topical eye drops, such as Tobradex®, can induce tear instability and decrease the number of mucin-expressing cells. [14], [25]–[27] Excessive instillation and incorrect use of preserved eye drops are important factors that contribute to the development of dry eye after phacoemulsification and corneal toxicity. However, the abnormal ocular surface staining in the current study showed typical interpalpebral staining pattern caused by dryness rather than inferior staining from drug toxicity or medicamentosa. The high incidence of abnormal Oxford Schema grading after phacoemulsification may be due to neurogenic inflammation.

Other factors associated with dry eye are older age, female gender, diabetes, [1], [4], [28]–[34] and systemic hypertension. [8], [35] However, in the current study; dry eye was not associated with those factors, which may have been due to the small sample size in the study. As there may be spontaneous appearance of dry eye, further study should be conducted to compare postoperative patients with subjects without operation who serve as control.

Although mild to moderate dry eye may not interfere with vision, decrease of vision can occur in severe cases. As a result, preoperative assessment should be done properly, Hardten [36] suggested using the ocular surface stress test, which takes about 30 to 60 minutes to perform and can be done after routine ocular examinations such as the slit-lamp examination and pupil dilatation. If an abnormal ocular surface is detected, the patients are at high risk of developing dry eye postoperatively. Other clinical tests such as the TBUT and fluorescein staining can be used to screen for dry eye. If dry eye is detected preoperatively, artificial tears or topical cyclosporine A (Restasis, Allergan, Irvine, California) can be prescribed postoperatively. [23].

We concluded that dry eye symptoms can develop immediately after phacoemulsification and the severity can peak on day 7. Both symptoms and signs of dry eye can improve over time. However, it is important that ophthalmologists assess dry eye before and after phacoemulsification to ensure proper treatment, quality of vision, and quality of life for their patients.

## What Was Known

* Dry eye can develop after various types of ophthalmic surgeries including cataract surgery.

* Phacoemulsification can induce dry eye-like syndrome and can also make dry eye symptoms worsening.

## What This Paper Adds

* The incidence of dry eye after phacoemulsification was 9.8%.

* Dry eye symptoms can develop immediately after phacoemulsification and the severity can peak on day 7. Both symptoms and signs can improve over time. The dry eye pattern was similar between all three clinical tests (TBUT, Schirmer I without anesthesia, Oxford ocular surface staining system) and the OSDI questionnaire.
